# Efficacy of Intense Pulsed Light in the Treatment of Recurrent Chalaziosis

**DOI:** 10.3389/fmed.2022.839908

**Published:** 2022-03-01

**Authors:** Yirui Zhu, Xiaodan Huang, Lin Lin, Mengshu Di, Ruida Chen, Jilian Dong, Xiuming Jin

**Affiliations:** ^1^Eye Center, Affiliated Second Hospital, School of Medicine, Zhejiang University, Hangzhou, China; ^2^Eye Department, Affiliated Dongyang Hospital, Wenzhou Medical University, Dongyang, China; ^3^School of Public Health, Nanjing Medical University, Nanjing, China

**Keywords:** recurrent chalaziosis, meibomian gland, IPL-MGX treatment, IPL, chalazion

## Abstract

**Purpose:**

High recurrence rate of chalaziosis and serious side effects of repeated surgical excision may help increase awareness of recurrent and refractory chalaziosis as a serious disorder affecting many aspects of life. This present study was aimed to investigate the efficacy and safety of intense pulse light (IPL) therapy and meibomian gland expression (MGX) in cases of recurrent chalaziosis after excision surgery.

**Methods:**

Forty-two consecutive recurrent chalaziosis cases (35 patients) treated with IPL-MGX were enrolled. All patients initially underwent excision with curettage. One week after lesion excision, IPL-MGX were performed at least 3 times. Another set of age- and sex-matched consecutive cases of recurrent chalaziosis, who received excision with curettage, but went without IPL-MGX treatment, were collected to calculate recurrence rate. Treatment efficacy and safety were measured before IPL-MGX treatment and 1 month after the final treatment.

**Results:**

The majority of patients received 4 sessions of IPL-MGX therapy (20 patients; 57.1%) or 3 sessions of IPL-MGX therapy (10 patients; 28.6%), resulting in a lower recurrence rate of 11.4% compared to that of recurrent chalaziosis without IPL-MGX cases (45.6%, *P* < 0.001). The NIBUT was significantly prolonged from 3.9 ± 1.8 to 5.1 ± 1.7 s at 4 weeks after the final treatment (*P* = 0.001). Similarly, mean TMH score improved and was statistically significant when compared with baseline (0.17 ± 0.07 vs. 0.21± 0.09; *P* = 0.008). Furthermore, meibum quality and expressibility scores significantly improved at 4 weeks following the final treatment (both *P* < 0.001). Other variables, such as intraocular pressure and visual acuity, remained unaffected following treatment.

**Conclusion:**

The combination of IPL treatment and MGX offers a low risk and effective option in decreasing the recurrence rate of recurrent chalaziosis by improving meibomian gland function. IPL-MGX may be considered for first-line treatment in recurrent or refractory cases post excision.

## Introduction

Chalazion is a common eyelid disease generally caused by blocked meibomian glands and chronic lipogranulomatous inflammation (1). After blockage, secreted lipids accumulate and erupt from the gland into the defensive collagen matrix of the tarsus. These lipids irritate and provoke a granulomatous inflammatory reaction resulting in the accumulation of immune cells, including polymorphonuclear leukocytes, lymphocytes, and plasma cells (2). This can also result in changes concerning cosmesis of the eyelid, ocular symptoms such as inflammation and irritation, or even disruption of vision resulting from mechanical ptosis and corneal astigmatism (3). Multiple factors are claimed in the pathogenesis of chalazion, such as constitutional atopic and seborrheic, hormonal, immunological, presence of irritable bowel disease, iatrogenic, infectious, mainly related to *Staphylococcus aureus* and *Cutibaterium acnes*, demodicosis, dysmetabolic factors such as vitamin A deficiency and diabetes (4–7). In previous studies, higher incidences and recurrence of chalaziosis have been observed in patients with chronic blepharokeratoconjunctivitis and meibomian gland dysfunction (MGD), as these patients have long-standing poor meibomian gland function that subsequently alters morphology (8).

Recurrent and refractory chalaziosis are usually associated with chronic blepharitis, acne rosacea, and meibomitis. Repeated surgical excision to remove these recurrent chalaziosis may create conjunctival-tarsal scar tissue and/or injure the ducts of meibomian glands, thus blocking lipids in the proximal ducts and ultimately resulting in more granuloma formation (9). Surgical intervention may also result in undesired appearance changes such as madarosis, eyelid margin deformation, and scarring of the skin. Additionally, improper management of the underlying cause of the infection potentially leads to recurrent infections, or to the development of other diseases. High recurrence rate and other serious side effects may help increase awareness of recurrent and refractory chalaziosis as a serious disorder affecting many aspects of life.

Chalazion often may be self-limiting, as is the case in 25-50% of cases, and can be resolved with warm compresses and/or medical treatment within 1-3 months of onset (10). Treatment options for persistent lesions include steroid injection, lesion excision with curettage, or total excision (11). Although steroid injection is considered simple to perform with effective results, serious unintended adverse effects have been reported in previous studies (11, 12).

Despite chalazion being a local lesion of an individual meibomian gland, patients with recurrent chalaziosis and multiple chalaziosis are more likely suffer from MGD (13). Therefore, concentrating on the management of the condition and the function and morphology of all the meibomian glands is more important than just treating chalaziosis alone. Intense pulsed light (IPL) is widely used to treat dermatological conditions, such as facial telangiectasia, facial rosacea, pigmented lesions, and excessive hair growth (14, 15). In recent years, ophthalmologists have studied the efficacy and safety of IPL treatment for dry eye and MGD. Several studies have reported that IPL and meibomian gland expression (MGX) treatment significantly improves meibomian gland secretion and quality, and lengthens tear film break-up time (TBUT) in dry and MGD (16, 17). We hypothesized that IPL treatment on the skin adjacent to eyelids after chalaziosis surgery would result in better meibomian gland functioning and a lower recurrence rate.

To date, the outcomes of the use of this technology for the management of chalaziosis recurrence have not been previously reported. The purpose of the present study was to investigate the efficacy of IPL-MGX in cases of recurrent chalaziosis after surgery.

## Patients and Methods

### Patients

This study was a retrospective, interventional, consecutive case series. All enrolled patients underwent a complete ophthalmologic examination, including the assessment of onset, duration and location of the chalaziosis before the recruitment. Inclusion criterion was as follows: (1) eyes diagnosed with recurrent chalaziosis (defined by a previously diagnosed chalaziosis eye that had completely resolved after treatment and had recurred either at the same site or at a different site) were enrolled. Recurrent chalazia or multiple chalazia associated with meibomian gland dysfunction is called chalaziosis. Notably, these recurrent chalaziosis failed to resolve after conservative treatment, including antibiotic ophthalmic ointments, steroid injection, and warm compresses; (2) were examined in the eye clinic of the Affiliated Second Hospital of Zhejiang University (Hangzhou, China) between July 1, 2020 and December 31, 2020, and (3) had received IPL-MGX treatment 1 week after lesion excision (Recurrent chalaziosis with IPL-MGX). Another set of age- and sex-matched consecutive cases of recurrent chalaziosis, who received excision with curettage but without IPL-MGX treatment, were collected to calculate recurrence rate (Recurrent chalaziosis without IPL-MGX).

The exclusion criteria included the following: (1) any ocular infection, allergy, intraocular inflammation, ocular surgery, or ocular trauma in the past 6 months; (2) any eyelid diseases or structural abnormality; (3) any systemic diseases that may lead to dry eye or MGD and (4) skin pigmented lesion in the treatment zone. This study was approved by the Institutional Review Board of the Affiliated Second Hospital of Zhejiang University. The sample size calculation for unmatched case-control study showed a power of 80 and alpha error of 0.05, for sample size of 42 in the cases (recurrent chalaziosis with IPL group) vs. 57 in the control (recurrent chalaziosis without IPL group).

### Procedures

Patients whose lesions had failed to respond to antibiotic ointments and/or warm compresses treatment underwent excision with curettage. First, the eyelid was infiltrated with 2-3 ml 2% lidocaine. It was then everted using a chalazion clamp. A single vertical incision was made at the point of the lesion, while all pus material was cleaned with a curette, and the lesion's capsule was removed. The eye was bandaged for 2 h with an eye patch after applying antibiotic ointment.

**Figure 1:**
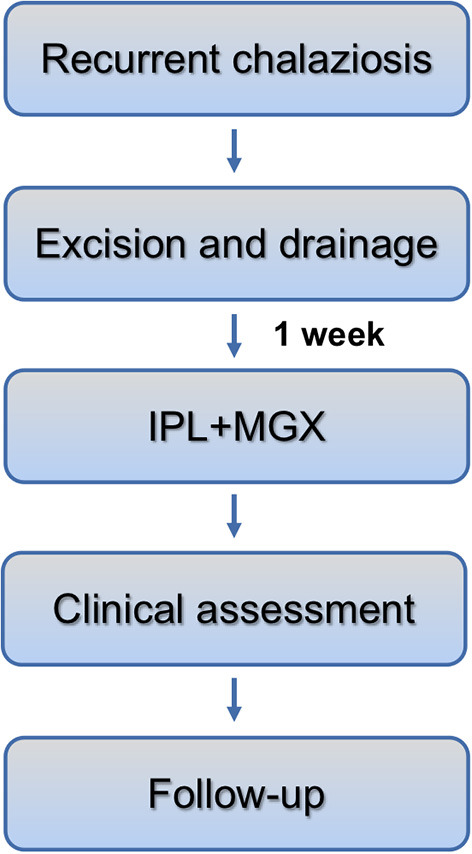
Time schedule of IPL-MGX in recurrent chalaziosis and clinical assessment of ocular surface.

One week after lesion excision, the E-Eye machine (E-SWIN company, France) IPL application was administered to the skin area below the lower eyelid (18). Briefly, the eyes were protected with opaque goggles, and ultrasound gel was applied to the patient's face from tragus to tragus. The intensity of the IPL treatment ranged from 9.8-13 J/cm^2^ in accordance with the Fitzpatrick Skin Type Grading. For each IPL treatment, five overlapping flashes were applied to the skin area below the lower eyelid with no pressure (19). After removal of the ultrasound gel, MGX was performed with a forceps-shaped meibomian gland compressor. The subjects received separate treatment sessions on days 1, 15, 45, and 75 per the manufacturer's recommendations until the lesion was resolved. All treatments and surgeries were performed by one ophthalmologist in the outpatient surgery room. All lesions were photographed before surgery and at each follow-up visit. The chalazion is considered to be resolved if the lesion size showed 80-100% regression, with no recurrence in 6 months, based on clinical evaluation and digital photographs (11). Patients whose lesion recurred were offered steroid injection or surgical excision and drainage (Figure 1).

### Clinical Assessment

To evaluate treatment efficacy, the following parameters were measured before the IPL-MGX treatment (baseline) and at 1 month after the final treatment. Non-invasive tear film breakup time (NIBUT), bulbar conjunctival hyperemia, and tear meniscus height (TMH) were assessed using the Keratograph 5M (Oculus, Wetzlar, Germany). The corneal fluorescein staining (CFS) score, meibum grade, and chalaziosis location were determined using slit-lamp microscopy. As previously reported, corneal fluorescein staining was measured using commercially available sterile fluorescein paper strips (Jinming New Technological Development Co. Ltd., Tianjin, China) (20). The corneal fluorescein staining score was assessed by grading the upper, middle, and lower parts of the cornea on a nine-point scale: no staining = 0; <5 stained punctate dots = 1; 5-9 stained punctate dots = 2; and ≥10 stained punctate dots or filamentous staining = 3. The total score for corneal fluorescein staining was calculated as the summation of all three parts of the cornea, and it ranged from 0 to 9 (21). The meibomian gland function was evaluated in accordance with the recommendations of the International Workshop on Meibomian Gland Dysfunction (22). The quality of the expressed meibum was scored as follows: clear = 1; cloudy = 2; granular = 3 and toothpaste = 4. The expressibility score of the meibum was assigned according to the secretory capacity percentage (Number of glands with secretory ability in the corresponding area the of chalazion/Total number of glands in the corresponding area) based on the International MGD Working Group Standard (all glands = 0; 60-80% of glands = 1; 20-40% of glands = 2; no glands = 3).

The safety of IPL-MGX treatment was evaluated by the measurement of visual acuity, intraocular pressure, lens opacity, as well as by fundus examination before and at 1 month and 6 months after the treatment session.

### Statistical Analysis

An independent sample *t*-test was used to evaluate differences in gender, age, duration of chalaziosis, and the presence of blepharitis and acne rosacea on the clinical outcome (IPL-MGX response and chalaziosis recurrence). Pearson bivariate correlation analysis was used to examine the influence of age, the duration of chalaziosis, the number of IPL-MGX, and chalaziosis recurrence. The recurrence rate values were compared with the χ2 test. SPSS 19.0 (SPSS, IBM Corporation, Chicago, IL, USA) was used for all analyses. A *P*-value of <0.05 was considered to be statistically significant.

## Results

**Figure 2:**
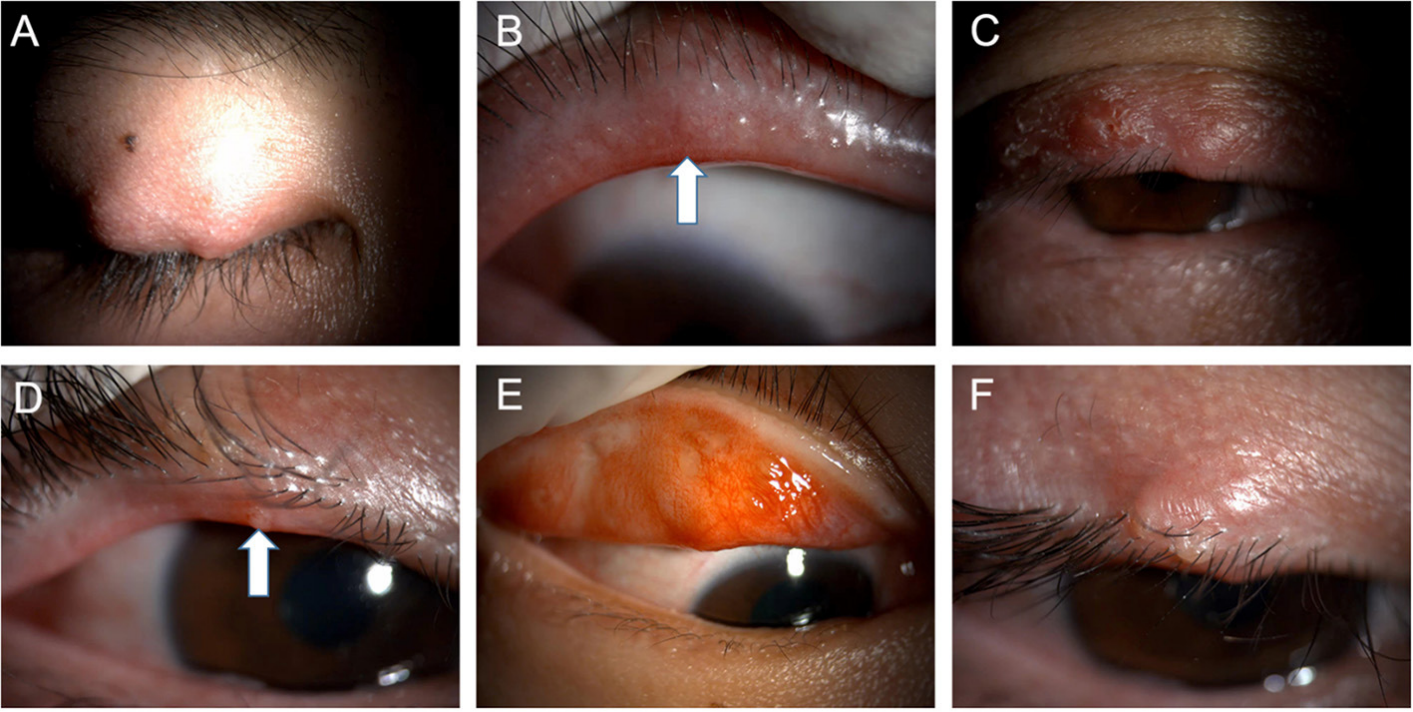
Typical images of the patients with recurrent chalaziosis or multiple chalaziosis during the pre-treatment stage **(A–F)**. Note the eyelid margin neovascularization **(B)** and lipid suppository **(D)** (white arrows) at the meibomian gland openings.

Forty-two consecutive recurrent chalaziosis eyes (35 patients, seven of whom had bilateral disease) were treated in the ocular disease clinic. Table 1 summarized the demographics and clinical characteristics of the patients. Most of the patients presented with recurrent chalaziosis (Figure 2). Of the recurrent chalaziosis eyes, 28.6% had secondary or multiple recurrences after a previous episode. The mean duration of the lesion was 2.3 months. Blepharitis was a common finding in 66.7% of the eyes. Acne rosacea was prevalent in 11.9% of the eyes.

**Table 1:** Demographic characteristics of recurrent chalaziosis.

**Characteristic**	**Recurrent chalaziosis with IPL**	**Recurrent chalaziosis without IPL**	***P*-value**
No. Eyes (patients)	42 (35)	57 (50)	-
**Gender**			0.73
Male	11 (31.5 %)	14 (28%)	-
Female	24 (68.5%)	36 (72%)	-
Age (years) (±SD; range)	38.5 ± 11.5 (24-74)	36.7 ± 14.1 (16-75)	0.18
Duration of chalaziosis (month, ±SD; range)	2.3 ± 1.8; 0.5-7	2.0 ± 1.6; 0.25-8	0.14
**Location**			0.13
Upper lid	21 (50%)	24 (42.1%)	-
Lower lid	6 (14.3%)	10 (17.5%)	-
Upper and lower lid	15 (35.7%)	12 (40.4%)	-
**Onset**			0.77
Second onset	30 (71.4%)	37 (64.9%)	-
Third onset	10 (23.8%)	16 (28%)	-
Multiple onset	2(4.8%)	4 (7.1%)	-
Blepharitis	28 (66.7%)	33 (57.9%)	0.37
Acne rosacea	5 (11.9%)	7 (12.3%)	0.95
**Previous treatment**			0.98
Lid hygiene	40 (95.2%)	57 (100%)	-
Topical antibiotic ointment	38 (90.5%)	52 (91.2%)	-
Excision	42 (100%)	57 (100%)	-

*SD, standard deviation*.

Most of the patients were treated previously with warm compresses and topical antibiotic ointment, and all eyes in current study underwent previous excision of the lesion. Twelve patients (34%) with recurrences had extruded contents sent to laboratory for histopathologic analysis after the second excision. All patients had normal histopathologic results without any evidence of cancer. There is no significance between chalaziosis with and without IPL-MGX cases in age, chalaziosis duration, location, onset, and previous treatment. However, the recurrence rate is significantly higher in recurrent chalaziosis without IPL-MGX cases (45.6%, *P* < 0.001).

**Figure 3:**
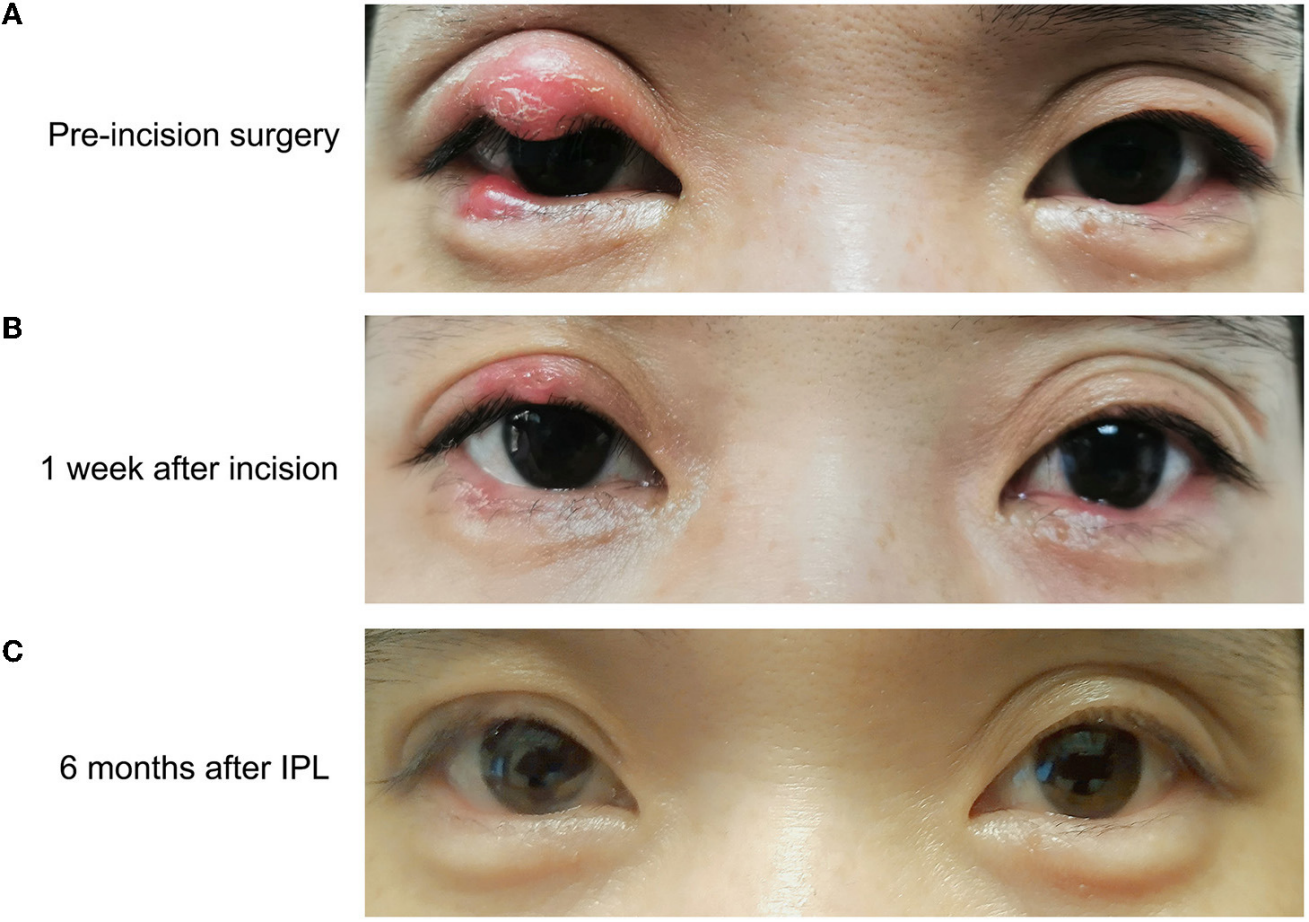
Bilateral upper and lower eyelid chalaziosis before incision surgery **(A)**, 1 week after incision **(B)** and 6 months after IPL-MGX therapy. Note complete resolution of the lesion by 6-months follow-up **(C)**.

The mean number of IPL-MGX treatments was 3.9 ± 0.8, with a range of 3-6 treatments. Most of the patients received four rounds of IPL-MGX therapy (20 patients; 57.1%), with others at three rounds of IPL-MGX therapy (10 patients; 28.6%), five rounds of IPL-MGX therapy (3 patients; 8.6%), or six rounds of IPL-MGX therapy (2 patients; 5.7%; Table 2, Figure 3). Recurrent lesions (4 patients; 11.4%) that failed to respond to two IPL-MGX treatments were recommended to undergo steroid injection or excision with curettage. No correlation was found between age, duration of chalaziosis, the number of IPL-MGX, and chalaziosis recurrence (Pearson bivariate correlation).

**Table 2:** Treatment outcome of recurrent chalaziosis in 35 consecutive patients (42 lesions).

	**Value**
**Number of IPL**
3	10 (28.6%)
4	20 (57.1%)
5	3 (8.6%)
6	2 (5.7%)
Average number of IPL (±SD)	3.9 ± 0.8
Chalaziosis recurrence	4 (11.4%)
Preoperative and postoperative VA	20/25, 20/25 (ns)
Preoperative and postoperative IOP (mmHg)	13.0, 14.5 (ns)
Follow up (month, ±SD; range)	7.19 ± 1.1; 6-10

*SD, standard deviation; VA, visual acuity; IOP, intraocular pressure; ns, not significant. Resolution was defined as at least 80% decrease in size with no recurrence*.

Visual acuity and intraocular pressure remained unchanged after IPL-MGX treatment. The average visual acuity was 20/25 before and after treatment, and the average intraocular pressure changed from 13.0 to 14.5 mmHg (paired samples *t-*test; not significant). No complications were encountered in the current study.

**Figure 4:**
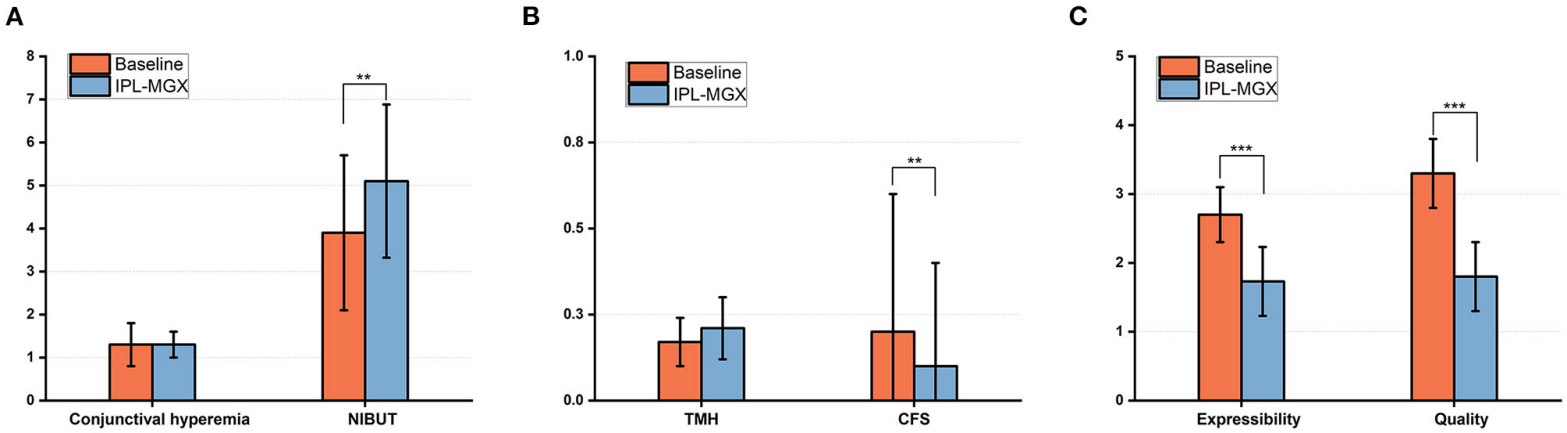
Changes in the bulbar conjunctival hyperemia, NIBUT **(A)**, TMH, CFS score **(B)**, and the expressibility and quality of meibum **(C)** between baseline and 4 weeks after the final IPL-MGX treatment session (**P < 0.01, ***P < 0.001).

The NIBUT (normal > 10 s) was significantly prolonged from 3.9 ± 1.8 to 5.1 ± 1.7 s at 4 weeks after the final treatment (*P* = 0.001). There was also an increase in the mean TMH score, which was statistically significant when compared with the baseline (0.17 ± 0.07 vs. 0.21 ± 0.09; *P* = 0.008). However, bulbar conjunctival hyperemia and corneal fluorescein staining decreases were not statistically significant (*P* = 0.8, *P* = 0.3; respectively; Figures 4A,B). Furthermore, the meibum quality and expressibility scores significantly decreased at 4 weeks after the final treatment (both *P* < 0.001, Figure 4C).

## Discussion

To our knowledge, this is the first study to show that IPL-MGX therapy following recurrent chalaziosis surgery significantly decreases the recurrence rate of chalaziosis. IPL-MGX treatment resulted in significantly better meibomian gland secretion function, improvements in dry eye symptoms, length in TBUT, and a very low recurrence rate after an average of four treatments. No adverse effects were attributed to IPL-MGX therapy. This study obtained new insight into the effect of IPL-MGX therapy on the recurrence rate of chalaziosis.

During excision with the curettage process, several meibomian glands were clogged with orifice-plugging meibum and toothpaste-like meibum. They were found in locations where recurrent chalaziosis were present before treatment, which suggests that local meibomian gland function had already changed before the chalaziosis formed and that the meibomian gland obstruction may have led to chalaziosis formation. This indicates that complete chalaziosis resolution does not indicate the end of the treatment. Once the meibomian gland function in the non-chalaziosis area does not improve, the meibomian glands would become obstructed resulting in the formation of chalaziosis again. This supports the observation that some people are more prone to recurrent chalaziosis. As the results showed, the recurrence rate of chalaziosis without meibomian gland treatment was significantly higher compared to the treated group (45.6 vs. 11.4%, respectively). One reason was that the improvement of meibomian gland function may differ between the two groups, since chalaziosis are closely related to the meibomian gland.

Conservative measures for treating chalaziosis include following proper eyelid hygiene, the use of warm compresses, and antibiotics. The initial treatment of chalaziosis is typically limited to the application of a topical antibiotic in conjunction with warm compresses. Antibiotics can be administered locally at the site of infection, or may be given systemically. Application of topical antibiotics may reduce the healing time by fighting against the causative bacterial infection and reducing inflammation (13). Tetracyclines are effective in the treatment of acne rosacea. Erythromycin lengthens tear break-up time and helps resolve punctate keratopathy by improving meibomian gland function (23). In up to 25% of onset cases, chalazia may resolve spontaneously within a mean duration of 6 months (24). However, a randomized multicenter study demonstrated that the complete resolution rates were low for each of these three conservative treatments. The overall complete resolution rate for the study was only 18%, with a range of 16-21%, depending on the specific treatment group (25). Advocating invasive therapy, such as excision with curettage or steroid injections, should be considered if the chalazia have been present for a long period of time.

Previous studies have evaluated the efficacy of steroid injections for chalazia (26). These studies confirmed that the use of intralesional triamcinolone acetonide was as effective as excision with curettage for the treatment of primary chalazia. Serious complications of intralesional steroid injection, such as retinal and choroidal vascular occlusion, inadvertent globe penetration and delayed post-injection hemorrhage have been described (12, 27, 28). These serious complications are rare, however, skin depigmentation changes can be more common at the injection site (29). Excision with curettage is recommended for the treatment of infected chalazia, larger lesions, and cases of recurrent chalazia that necessitate biopsy. Treatment by excision and curettage is simple and less inconvenient, but the eyelid may be somewhat painful. Surgery is a less favorable option for younger age patients who would not tolerate a longer surgery and patients who may have substantial psychological fear of surgery (30). Moreover, multiple and marginal chalaziosis excision may result in permanent eyelid functional and aesthetic defects. IPL-MGX therapy is comfortable and, in most cases, would be more likely to be accepted by younger age and older age patients as opposed to surgery.

Few studies have reported the recurrence rate of refractory and recurrent chalaziosis after clinical treatments. A meta-analysis compared the efficacy of excision with curettage and intralesional steroid injections for chalazia treatment showing that the recurrence rate ranged from 0 to 16.7% for excision with curettage and 0-27.3% for intralesional steroid injections. These results show that the recurrence rates after both treatments are low since most of the primary chalazia were enrolled. Despite the high prevalence of recurrent chalaziosis in clinical practice, there is few study reporting the recurrence rate of recurrent chalaziosis after various treatments. In our study, we compared the recurrence rate of recurrent chalaziosis after second surgery with and without IPL-MGX therapy. IPL-MGX treatment significantly improved meibomian gland function and decreased the recurrence rate of chalaziosis.

Chalazion patients commonly present with coexisting blepharitis or acne rosacea, which are associated with telangiectasias and superficial angiogenesis that produce inflammatory mediators that may travel to the eyelids (31). Several studies have reported that IPL-MGX treatment can relieve dry eye symptoms in refractory MGD cases (16, 17, 31). Studies on IPL use for acne vulgaris have shown a reduction in inflammatory infiltrates around the area of meibomian glands and sebaceous glands (32, 33). Furthermore, a high prevalence of MGD has been evidenced in patients with the autoimmune disease Sjögren's syndrome. A toxic environment is created by the conjunctival inflammation with the lymphocyte accumulation leading to tarsal and peri-glandular inflammation (34). It was demonstrated that the IPL treatment reduce the ocular discomfort and improves ocular surface signs and symptoms (35). Both tear film stability and meibomian gland function responded positively to IPL-MGX in our study, resulting in improvement in the condition of tear film stability and meibomian secretion. These results may explain the IPL-MGX-induced lower recurrence rate of recurrent chalaziosis. Possible mechanisms of underlying the effects of IPL-MGX treatment in recurrent chalaziosis include the inhibiton of superficial angiogenesis that decrease inflammatory infiltration, reduction of microorganisms on the eyelids and thermal effect of IPL facilitating meibomian gland secretion (36, 37).

Limitations of our study included potential bias due to surgeon variation in technique and previous surgical experience on initial chalazia. In order to minimize variation, a fixed surgeon was chosen to perform excision with curettage on the recurrent chalaziosis. Secondly, the single-arm study was designed based on a small sample size. A larger sample size of recurrent chalaziosis patients and a more controlled experimental design are will be preferable for future studies. Third, patients' conservative treatments varied prior to being enrolled in the study. Furthermore, dry eye and meibomian function tests were not observed in the recurrent chalaziosis without the IPL-MGX group. There were other inconsistencies, such as the variation in number of IPL-MGX treatments, which depended on the number of chalaziosis, the chalaziosis size, the lesion duration, and the patient's consent.

In summary, the management of recurrent or multiple chalaziosis in clinical practice remains challenging as the conservative therapies are notoriously poor. We found that IPL treatment combined with MGX is effective and safe in decreasing the recurrence rate of chalaziosis by promoting meibomian gland function. Our results present the possibility of an important new approach for treatment of recurrent or refractory chalaziosis due to meibomian gland function. Further studies are required to determine if the first-line choice of IPL-MGX treatment improves the outcome of specific types of refractory chalaziosis in clinical scenarios.

## Data Availability Statement

The original contributions presented in the study are included in the article/supplementary material, further inquiries can be directed to the corresponding author/s.

## Ethics Statement

The studies involving human participants were reviewed and approved by the Institutional Review Board of the Affiliated Second Hospital of Zhejiang University. The patients/participants provided their written informed consent to participate in this study. Written informed consent was obtained from the individual(s) for the publication of any potentially identifiable images or data included in this article.

## Author Contributions

YZ: study concept, design, and write the manuscript. LL: perform surgery and treatment. MD and RC: data collection. YZ, XH, MD, RC, and JD: analysis and interpretation of data. YZ, XH, and XJ: critical revision of the manuscript. XJ: supervision. All authors read and approved the final manuscript.

## Funding

The authors acknowledge the financial support of the National Natural Science Foundation of China (Project Number: 81870624) and the Natural Science Foundation of Zhejiang Province (LQ20H120008).

## Conflict of Interest

The authors declare that the research was conducted in the absence of any commercial or financial relationships that could be construed as a potential conflict of interest.

## Publisher's Note

All claims expressed in this article are solely those of the authors and do not necessarily represent those of their affiliated organizations, or those of the publisher, the editors and the reviewers. Any product that may be evaluated in this article, or claim that may be made by its manufacturer, is not guaranteed or endorsed by the publisher.
